# Position estimation with a millimeter-wave massive MIMO system based on distributed steerable phased antenna arrays

**DOI:** 10.1186/s13634-018-0553-9

**Published:** 2018-06-05

**Authors:** Nenad Vukmirović, Miloš Janjić, Petar M. Djurić, Miljko Erić

**Affiliations:** 1 School of Electrical Engineering, University of Belgrade, Belgrade, Serbia; 2 Innovation Center of School of Electrical Engineering, University of Belgrade, Belgrade, Serbia; 3 Department of Electrical and Computer Engineering, Stony Brook University, New York City, NY USA

**Keywords:** Direct position estimation, mmWave, Massive MIMO, Steerable phased antenna arrays, Wireless indoor localization

## Abstract

In this paper, we propose a massive MIMO (multiple-input-multiple-output) architecture with distributed steerable phased antenna subarrays for position estimation in the mmWave range. We also propose localization algorithms and a multistage/multiresolution search strategy that resolve the problem of high side lobes, which is inherent in spatially coherent localization. The proposed system is intended for use in line-of-sight indoor environments. Time synchronization between the transmitter and the receiving system is not required, and the algorithms can also be applied to a multiuser scenario. The simulation results for the line-of-sight-only and specular multipath scenarios show that the localization error is only a small fraction of the carrier wavelength and that it can be achieved under reasonable system parameters including signal-to-noise ratios, antenna number/placement, and subarray apertures. The proposed concept has the potential of significantly improving the capacity and spectral/energy efficiency of future mmWave massive MIMO systems.

## Introduction

Millimeter-wave (mmWave) communication and massive MIMO (multiple-input-multiple-output) are disruptive technologies for cellular 5G (5th generation) systems. Not surprisingly, they have been in the focus of intensive research efforts in both academia and industry in the last decade. The application of massive MIMO systems in the mmWave band represents a big research and technological challenge. Since the work of Marzetta in 2010 [1], there have been many technical papers on this technology. Some address system issues [2–6], and others signal processing [7], analog and hybrid beamforming [8–17], propagation and channel modeling/measurement [18–20], technological aspects [21], and practical implementations [22, 23].

Multi-user MIMO systems referred to as massive MIMO systems were introduced in [1]. Unlike in conventional MIMO systems for point-to-point communications where the channels between pairs of antennas are assumed uncorrelated, in massive MIMO systems there is a large number of antennas at a base station (BS). The antennas of the system form beams toward low-cost user devices with spatially separated single antennas [1]. Many antennas are required in the mmWave band because of the high pathloss and the need for large antenna gains to obtain sufficiently high signal-to-noise ratios (SNRs).

Traditionally, beamforming by the antennas is realized completely in the digital domain. This entails that every antenna has its own radio-frequency (RF) chain (a low-noise amplifier, a down-converter, an A/D converter at the receiving side, a D/A converter, up-converter, and a power amplifier at the transmitting side), which renders the application of massive MIMO in mmWave impractical due to high cost and energy consumption. A promising solution to these problems lies in the concept of hybrid transceivers, which use a combination of analog beamforming in the RF domain and digital beamforming in the baseband to allow for RF circuits with a smaller number of up/down conversion chains. In practice, a beamformer is usually implemented as an array of phase shifters with only a discrete set of possible shifts (phase quantization) [7]. Interest in hybrid transceivers has accelerated over the past 3 years (especially following [7]), and as a result, various structures have already been proposed.

In the wide literature, there are only a few papers dealing with localization with mmWave massive MIMO systems. The authors of [24] surveyed applications of localization in massive MIMO systems and state that the 5G technology is expected to allow localization accuracy of 1 cm, which is twice the carrier wavelength at 60 GHz (around 5 mm). In [25], high-accuracy localization with mmWave systems in applications related to assisted living and location awareness was considered. It was concluded that “future 5G mmWave communication systems could be an ideal platform for achieving high-accuracy indoor localization.” The performance of localization based on the RSSI (received signal strength indicator) principle applied to the mmWave range was investigated in [26], and it was found that it was possible to achieve accuracy of around 1 m. A fingerprint-based localization was presented in [27], and a method for direct localization was introduced in [28]. In [29], the authors presented an mTrack system for high precision passive object tracking at 60 GHz and claimed that submillimeter accuracy could be achieved. This accuracy can provide location awareness in massive MIMO systems that can be exploited to improve communication and enable location-based services. Performance limits of localization by beamforming with mmWave systems was studied in [15]. The problem of positioning and orientation of subarrays of user nodes was investigated in [30, 31]. Papers [32] and [33] propose a method for localization/tracking of moving terminals in dense urban environments in 5G based on intermediate ToA/DoA (time of arrival/direction of arrival) estimates at base stations. The method consists of two steps and is implemented using extended Kalman filters and achieves sub-meter accuracy in cmWave. This error is about five times larger than the carrier wavelength but is sufficient for location aware communications [24]. In [34], a solution to non-cooperative transmitter localization is presented. The solution is based on sectorized antennas and intermediate DoA and RSS (received signal strength) estimates at base stations. That paper also provides the CRBs (Cramér-Rao bounds) for DoA/RSS and localization errors, and it shows that the methods achieve sub-meter accuracy.

One may argue that localization, especially in coherent LoS (line-of-sight) scenarios (typical of the mmWave band), can have profound implications on system capacity. Namely, if it is possible to localize a UT (user terminal) with an accuracy much better (by two orders of magnitude) than the carrier wavelength, then it is conceivable to focus energy from distributed transmitters to the location of the UT (and to possibly other locations, if there are more users) with greatly reduced interference levels to other users. This clearly suggests that accurate location awareness enables location-aided communication.

Our previous research has confirmed that in a spatially coherent scenario (where the LoS component is dominant and where the carrier phase changes predictably over distance), a distributed antenna array and direct localization algorithms can achieve localization accuracy much better than the carrier wavelength (by two to three orders of magnitude). In [35], it was reported that accuracy of 30% of carrier wavelength in RFID (radio frequency identification) localization was achieved. Localization in a spatially coherent scenario was also addressed in [36, 37]. The spatially coherent approach suffers from high side lobes in the criterion function (localization ambiguity). This problem of side lobes is similar in nature to the one of side/grating lobes in direction of arrival estimation with classical antenna arrays.

In this paper, we aim at achieving a high localization accuracy with distributed antenna subarrays in mmWave, where the accuracy would be much better than the carrier wavelength. At the same time, we also resolve the problem of localization ambiguity. New research problems arise with this including designing an architecture of such system and formulating algorithms which achieve these two goals. Even though the focus of this paper is localization with mmWave massive MIMO systems, the proposed localization algorithms are applicable to cmWave bands as well. This is an important feature of the algorithms because the 3GPP (3rd Generation Partnership Project) group has started to define bands for 5G and the cmWave bands are expected to be used in the first phase of 5G networks [38]. The contributions of our paper are as follows:

1. We propose an innovative mmWave massive MIMO architecture for accurate localization. In the proposed concept, the BS uses distributed “subarray units,” which are connected to the fusion center of the BS by calibrated wired or fiber-optic links. Each subarray unit has one “omni antenna” and one phased antenna subarray (thus, there are two RF chains in total). The distributed array composed of omni antennas is used for the detection of signal presence (interception), estimation of time axes misalignment between the UT and BS, and accurate coherent localization. The antenna subarrays are used to estimate the location of the UT once its presence has been detected.
2. We propose coherent and non-coherent localization algorithms. The algorithms are of maximum likelihood (ML)-type for single user and additive white Gaussian noise scenarios, but can also be applied to multi-user settings because they are user-selective (a user’s code sequence is adopted in the criterion functions of the algorithms).
3. We formulate a multistage/multiresolution searching and scanning strategy to achieve high localization accuracy, which is much better than the carrier wavelength. The strategy also circumvents the ambiguity problem. The idea is to split the localization process into stages in which increasingly accurate estimates are made over smaller and smaller domains.

In the paper, we also demonstrate the performance of the proposed algorithms with extensive simulations. The numerical experiments were carried out to study the performance in LoS-only and multipath (LoS + NLoS) scenarios.

The rest of this paper is organized as follows. Section 2.1 introduces the system architecture of the mmWave massive MIMO system with distributed subarrays, the multistage/multiresolution searching and scanning strategy for localization, and the mathematical models of the signals. In Section 2.2, we propose three different classes of algorithms for multistage/multiresolution searching and scanning. In Section 3, we demonstrate the performance of the system and the methods with Monte Carlo simulations, and we discuss the obtained results. Concluding remarks are given in Section 4.

## Methods

The aim of the research is to develop algorithms for coherent passive localization in massive MIMO systems with distributed phased antenna arrays, so that the localization error is a small fraction of the carrier wavelength, and also to solve the ambiguity problem, inherent to coherent position estimation. We have proposed an innovative model of a massive MIMO system with distributed phased antenna arrays, formulated a signal model for this system model, proposed a multistage/multiresolution localization strategy, and proposed new localization algorithms. The performance of the proposed strategy and its algorithms is evaluated by running Monte Carlo simulations in which the signals were generated according to the proposed system and signal models, and then the location of the simulated transmitter was estimated using the proposed strategy.

### System model of mmWave massive MIMO with distributed subarrays

#### System architecture

**Fig. 1:**
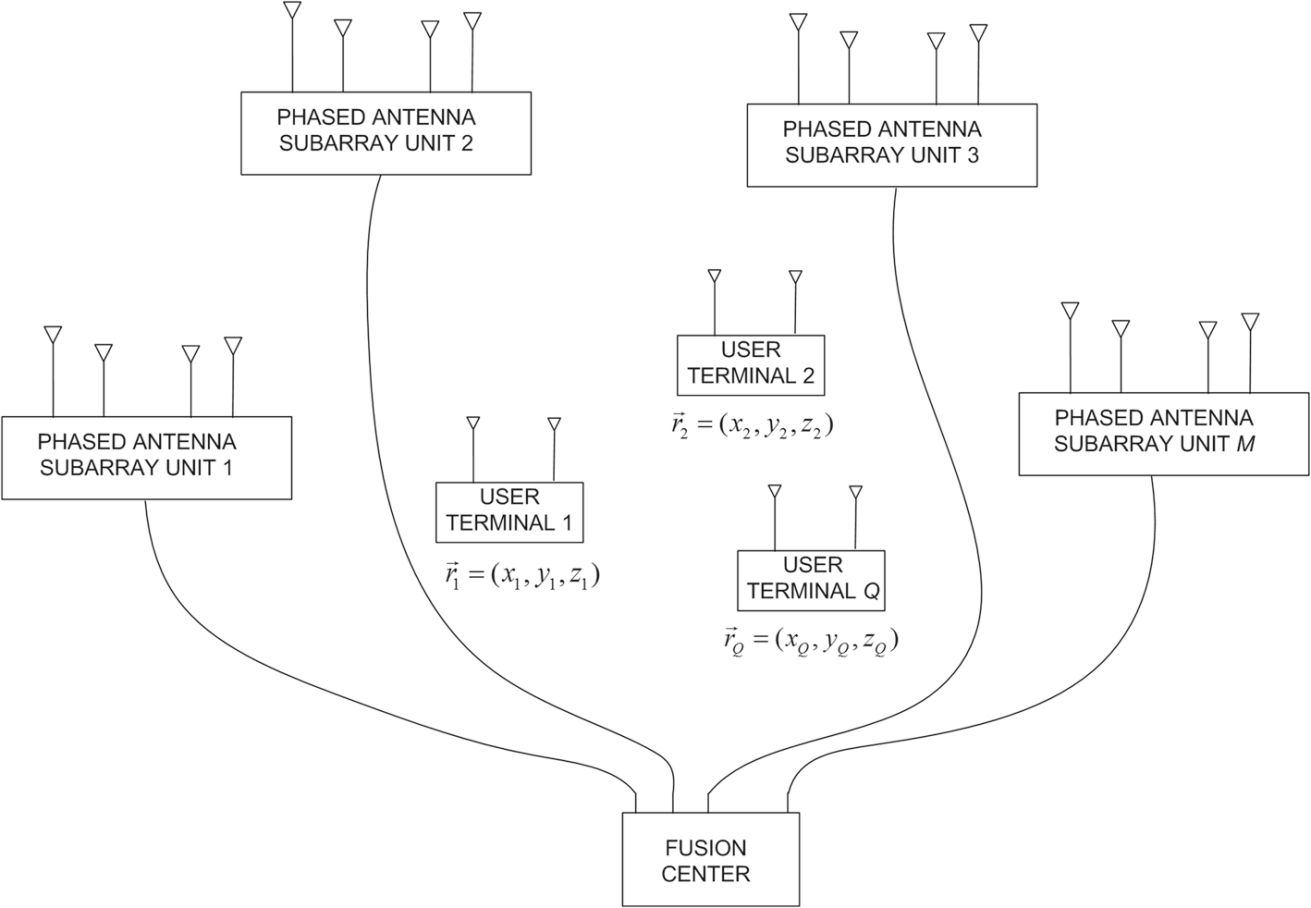
The system architecture

Our system uses a distributed antenna array to selectively estimate the position of an independent RF transmitter, Tx, based on its code sequence (known to the system). All the antennas, including the transmitting one, are distributed indoors and are either stationary or slow-moving (see Fig. 1). The slow-moving requirement is needed to allow for neglecting Doppler effects. The receiving antennas are grouped in *M* “subarrays.” The distances between the antennas within the same subarray are of the order of the carrier wavelength, *λ*_*c*_.

**Fig. 2:**
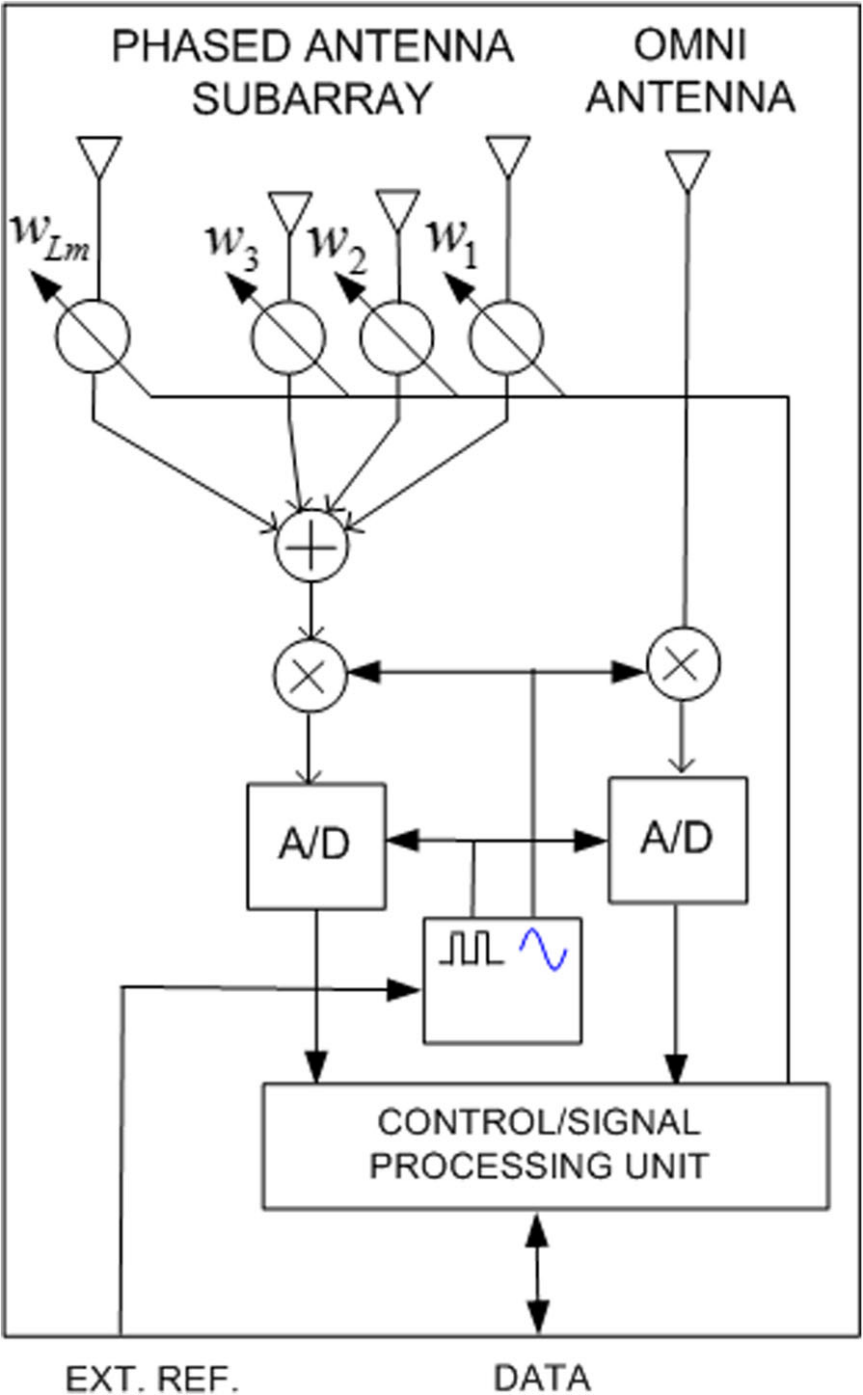
A phased antenna subarray unit

The *m*th subarray has *L*_*m*_ antennas with positions $\vec {r}_{m,l}\,=\, \left (x_{m,l},y_{m,l},z_{m,l}\right)^{\top }\!$, *m* ∈ {1,2,…,*M*}, and *l* ∈ {1,2,…,*L*_*m*_}. The signals from those antennas are inputs to a beamformer, which multiplies them by complex coefficients *w*_*m,l*_ that are electronically set in advance (see Fig. 2). The output of the *m*th beamformer is IQ (in-phase quadrature-phase) demodulated and A/D (analog-to-digital) converted to obtain the (complex) samples of the *m*th channel. Further, each subarray has an omnidirectional receiving antenna at $\vec {r}_{m,0}=\left (x_{m,0},y_{m,0},z_{m,0}\right)^{\top }$ with its own A/D converter. Thus, the digital signal processor (DSP) in the fusion center has access to 2*M* channels.

Another option is to have A/D converters and processing circuitry at the units. Then, they are digitally connected to the fusion center.

The Tx antenna is at an unknown position $\vec {r} = \left (x,y,z\right)^{\top }$, whereas the three-dimensional positions of all the other antennas in the system are known. All the receiving channels are time, phase, and frequency synchronized to each other. Time synchronization between the Tx and our system is not required. However, it is assumed that they both use the same (known) carrier frequency. To perform the most accurate position estimation, each of the channels, including the one of the Tx, must match the phase of its local carrier to its clock. With the matching, the carrier phase would be 0 at each beginning of observation interval.

In summary, every antenna unit in the proposed system includes one omni antenna and one phased antenna array (two receiving channels are needed at each antenna unit). Thus, we have two functionally independent, mutually synchronized distributed antenna systems in time and frequency.

#### Multistage/multiresolution searching and scanning strategy

**Fig. 3:**
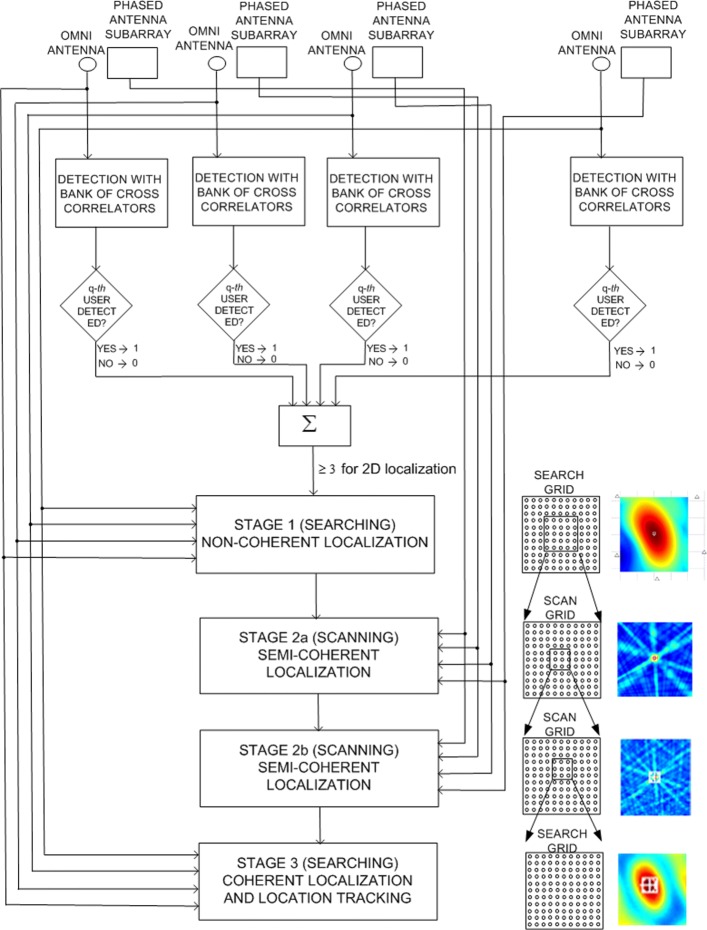
The block scheme of the multistage/multiresolution search strategy

The system performs detection and location estimation of user transmitters in three stages, Fig. 3. In stage 1, the system runs a numerically low-intensive algorithm to detect the presence of RF transmissions and to obtain approximate estimates of the transmitters’ locations. Only the omni antennas are employed in stage 1, and they can be used all the time. To start the estimation, the algorithm has to wait only for a single period of the Tx sequence. Each omni antenna channel has a bank of as many cross-correlators as there are user sequences of interest. When at least three cross-correlators detect the presence of a sequence **s** for two-dimensional localization (or four for three-dimensional localization), the algorithm performs coarse localization of this user (with sequence **s**) over a grid that spans the entire area of interest. The resulting inaccuracy of the estimated locations is expected to be of the order of 10*λ*_*c*_ or more.

In stage 2, another algorithm refines the search of the previous stage by scanning the area around the previous estimates using the subarrays. Since each subarray can only operate with a single set of coefficients *w*_*m,l*_ at a time, more than one observation period is needed for a single estimate. The length of the period corresponds to the period of the user sequence. Also, there must be time intervals between the periods so that the beamformers can change their coefficients.

Stage 2 can be split into steps 2a, 2b, etc., each corresponding to beamformers with different beam widths, resolutions, and scan areas. The number of steps depends on the ratio of the resulting root-mean-squared error (RMSE) of stage 1 and the required RMSE of stage 2. The larger the ratio, the more steps should be used to keep the number of observation intervals down. The coefficients in stage 2a are chosen to create relatively wide (sector) beams for the subarrays in order to decrease the number of points on the scan grid, while still providing an SNR (signal-to-noise-ratio) gain compared to that of the omnidirectional antennas. This translates into a smaller number of sequence periods required for estimation.

The last step of stage 2 uses the narrowest possible beams for the given subarrays, and it scans the smallest area. Each scan point requires a new sequence period. The scan grid needs to be sufficiently fine so that the resulting location error is below *λ*_*c*_. The overall purpose of this stage is to shrink enough the search area so that in the third stage one can solve the so called ambiguity problem, discussed later in the text, which is inherent to the applied algorithm.

In stage 3, only one sequence period is needed and only the signals from the omni antennas are used. The algorithm in this stage relies on the phase relations among the different channels to make the most accurate estimates. The search grid is small but very fine because the resulting error is expected to be of the order of *λ*_*c*_/100 or better. When the Tx is localized with this accuracy and it moves, it can be tracked by continuously running the same algorithm.

#### Signal model

The Tx prepares a periodic training signal in the following way. A complex sequence **s**=[*s*_0_,*s*_1_,…,*s*_*N*−1_]^⊤^, assigned to a user, is repeated multiple times and D/A (digital-to-analog) converted with sampling frequency *ν*_*s*_. The resulting periodic continuous-time signal is *s*(*t*), where the time variable *t* in the mathematical model is normalized with 1/*ν*_*s*_. For compatibility between the discrete-time and continuous-time domains, we use normalization of time values with 1/*ν*_*s*_ and frequencies with *ν*_*s*_ throughout the paper. The real and imaginary components of *s*(*t*) are upconverted to the carrier frequency *ν*_*c*_ with quadrature carriers. The resulting RF signal is periodic with period *N*/*ν*_*s*_ and its bandwidth is *B*. The signals in all the channels are sampled at the Nyquist rate, which implies *B*=*ν*_*s*_.

The RF signal of the Tx propagates at *c*=3×10^8^ m/s. The *l*th antenna in the *m*th subarray receives the signal whose baseband equivalent is

1 $$\begin{array}{*{20}l} u_{m,l}(t) &= s_{m,l}(t) + \eta_{m,l}(t), \end{array} $$

2 $$\begin{array}{*{20}l} s_{m,l}(t) & = a_{m,l}e^{-j\omega_{c}\left(t_{0}+\tau_{m,l}\right)}s\left(t-t_{0}-\tau_{m,l}\right),  \end{array} $$

where *m*∈{1,2,…,*M*}, *l*∈{0,1,…,*L*_*m*_}. The index *l*=0 denotes the omni antenna associated with the appropriate subarray; *a*_*m,l*_ is an unknown real-valued attenuation coefficient; *ω*_*c*_=2*πf*_*c*_ and *f*_*c*_=*ν*_*c*_/*ν*_*s*_ are normalized carrier frequencies in radians per sample and cycles per sample, respectively; *t*_0_ is an unknown delay of the start of the transmission of a period of the Tx signal relative to the receiving system’s time axis; *τ*_*m,l*_=*d*_*m,l*_*ν*_*s*_/*c* is the propagation delay from the Tx to the appropriate receiving antenna where $d_{m,l} = \lVert \vec {r}-\vec {r}_{m,l}\rVert $; *η*_*m,l*_(*t*) is independent complex Gaussian noise in the frequency range (−1/2,1/2). The baseband equivalent of the signal at the output of the *m*th beamformer is

3 $$\begin{array}{*{20}l} u_{m}(t) & = s_{m}(t) + \eta_{m}(t), \end{array} $$

4 $$\begin{array}{*{20}l} s_{m}(t) &= \sum\limits_{l=1}^{L_{m}} w_{m,l}a_{m,l}e^{-j\omega_{c}\left(t_{0}+\tau_{m,l}\right)}s\left(t-t_{0}-\tau_{m,l}\right), \end{array} $$

5 $$\begin{array}{*{20}l} \eta_{m}(t) & = \sum\limits_{l=1}^{L_{m}} w_{m,l}\eta_{m,l}(t).  \end{array} $$

The DSP has access to the samples *u*_*m*_(*n*) and *u*_*m*,0_(*n*) for *m*∈{1,2,…,*M*} and *n*∈{0,1,…,*N*−1}.

The discrete-time matrix baseband model derived from (1)–(5) is given by

6 $$\begin{array}{*{20}l} \mathbf{u}_{m,0} &= \mathbf{s}_{m,0} + \boldsymbol{\eta}_{m,0}, \end{array} $$

7 $$\begin{array}{*{20}l} \mathbf{s}_{m,0} & = a_{m,0}\mathbf{F}^{\mathrm{H}}\mathbf{D}_{t_{0}+\tau_{m,0}}\mathbf{F}\mathbf{s}, \end{array} $$

8 $$\begin{array}{*{20}l} \mathbf{u}_{m} &= \mathbf{s}_{m} + \boldsymbol{\eta}_{m}, \end{array} $$

9 $$\begin{array}{*{20}l} \mathbf{s}_{m} &= \sum\limits_{l=1}^{L_{m}} w_{m,l}a_{m,l}\mathbf{F}^{\mathrm{H}}\mathbf{D}_{t_{0}+\tau_{m,l}}\mathbf{F}\mathbf{s}, \end{array} $$

where

10 $$\begin{array}{*{20}l} \mathbf{u}_{m,0} & = \left[u_{m,0}(0),u_{m,0}(1),\hdots,u_{m,0}(N-1)\right]^{\top}, \end{array} $$

11 $$\begin{array}{*{20}l} \boldsymbol{\eta}_{m,0} &= \left[\eta_{m,0}(0),\eta_{m,0}(1),\hdots,\eta_{m,0}(N-1)\right]^{\top}, \end{array} $$

12 $$\begin{array}{*{20}l} \mathbf{u}_{m} & = \left[u_{m}(0),u_{m}(1),\hdots,u_{m}(N-1)\right]^{\top}, \end{array} $$

13 $$\begin{array}{*{20}l} \boldsymbol{\eta}_{m} &= \left[\eta_{m}(0),\eta_{m}(1),\hdots,\eta_{m}(N-1)\right]^{\top}, \end{array} $$

are all *N*×1 complex vectors, **D**_*τ*_ is a time-delay-by- *τ* operator that also models the appropriate carrier phase shift, and **F** is a modified DFT (discrete Fourier transform) matrix such that **F**^−1^=**F**^H^ and whose rows are sorted by their corresponding natural RF frequencies. More formally,

14 $$\begin{array}{*{20}l} \mathbf{D}_{\tau} &= e^{-j\omega_{c}\tau}\text{Diag}\left\{\text{exp}\left(-j\frac{2\pi}{N}\tau\mathbf{k}\right)\right\}, \end{array} $$

15 $$\begin{array}{*{20}l} \mathbf{F} &= \frac{1}{\sqrt{N}}\text{exp}\left(-j\frac{2\pi}{N}\mathbf{k}\cdot\mathbf{n}^{\top}\right), \end{array} $$

where $\mathbf {n} = \left [0,1,\hdots,N-1\right ]^{\top }, \mathbf {k} = \left [-\frac {N}{2},-\frac {N}{2} +1,\hdots, \frac {N}{2}-1\right ]^{\top }$, exp() is the element-by-element exponential function, and Diag{} is a diagonal matrix with the given elements on its main diagonal.

### Direct position estimation algorithms

In this subsection, we describe algorithms for estimating the position of a user with a code sequence **s**, where the algorithms have different levels of accuracy and numerical complexity. The algorithms are derived for a single-user scenario; however, if the code sequences of the other users are orthogonal to **s**, the algorithms can also be applied in multi-user settings. If the sequences are not orthogonal and the users are sufficiently separated from each other in space, the algorithms should still work well.

#### Coherent algorithms

First, we discuss coherent algorithms, which rely on differences of carrier phases among signals from different channels and on differences of complex envelopes. We point out that information about the Tx location is also present in the signal amplitudes; however, we will not use it here.

The coherent algorithms only use the signals from the omni antennas; therefore, the available data for processing include the time samples *u*_*m*,0_(*n*) for 1≤*m*≤*M*, 0≤*n*≤*N*−1. We assume that the noises in the channels have the same power, which is known, so that *η*_*m*,0_(*n*) has a circularly symmetric Gaussian probability density function (PDF) with mean 0 and variance *σ*^2^, or $\eta _{m,0}(n) \sim \mathcal {CN}\left (0,\sigma ^{2}\right), \forall m$. In practice, if the noisy data have different powers, they can be scaled by different factors to make this condition hold. The PDF of the observed data is

16 $$\begin{array}{*{20}l} f_{\mathrm{C}}(\mathbf{u}_{0})&\propto \prod\limits_{m=1}^{M} \exp \left(-\lVert \mathbf{u}_{m,0} - \mathbf{s}_{m,0}\rVert^{2}/\sigma^{2}\right),  \end{array} $$

where ∥·∥ denotes the Frobenius norm. We want to estimate the unknown parameters of **s**_*m*,0_, ∀*m*, from which we can estimate the location of Tx.

According to the ML method, we maximize the likelihood function (also given by (16)) with respect to the unknown parameters, (*a*_1,0_,…,*a*_*M*,0_,*t*_0_,*x,y,z*). This maximization is equivalent to the minimization of $\sum _{m=1}^{M} \lVert \mathbf {u}_{m,0} - \mathbf {s}_{m,0}\rVert ^{2}$, or more specifically of

17 $$\begin{array}{*{20}l} g_{1}&= \sum\limits_{m=1}^{M} \left(a_{m,0}^{2}\lVert \mathbf{s}\rVert^{2} - 2a_{m,0}\text{Re}\left(\mathbf{u}_{m,0}^{\mathrm{H}}\mathbf{F}^{\mathrm{H}}\mathbf{D}_{t_{0}+\tau_{m,0}}\mathbf{F}\mathbf{s}\right)\right).  \end{array} $$

Note that the propagation times *τ*_*m*,0_ implicitly depend on the coordinates of the Tx, *x*, *y*, *z*.

The minimization can be first carried out over *a*_*m*,0_ (∀*m*) and then over (*t*_0_,*x,y,z*). For given *t*_0_, *x*, *y*, *z*, the ML estimate of *a*_*m*,0_ is given by

18 $$ {\begin{aligned} \widehat{a}_{m,0} & \,=\,\! \underset{a_{m,0}\in\left[0,+\infty\right)}{\mathrm{arg\,min}} \left(a_{m,0}^{2}\lVert \mathbf{s}\rVert^{2} \!\,-\, 2a_{m,0}\text{Re}\left(\mathbf{u}_{m,0}^{\mathrm{H}}\mathbf{F}^{\mathrm{H}}\mathbf{D}_{t_{0}+\tau_{m,0}}\mathbf{F}\mathbf{s}\right)\right)\\ &\!= \max\left\{0,\frac{1}{\lVert\mathbf{s}\rVert^{2}}\text{Re}\left(\mathbf{u}_{m,0}^{\mathrm{H}}\mathbf{F}^{\mathrm{H}}\mathbf{D}_{t_{0}+\tau_{m,0}}\mathbf{F}\mathbf{s}\right)\right\}.  \end{aligned}}  $$

Note that negative values are not allowed for the amplitude *a*_*m*,0_ and that the function being minimized is a second-degree polynomial of *a*_*m*,0_. After substituting (18) in (17), we obtain the estimates of *t*_0_,*x,y*, and *z* from

19 $$ {\begin{aligned} \left(\widehat{t}_{0},\widehat{x},\widehat{y},\widehat{z}\right) \,=\, \underset{t_{0},x,y,z}{\mathrm{arg\,max}} \sum\limits_{m=1}^{M} \!\!\left(\max\left\{\!0,\text{Re}\!\left(\mathbf{u}_{m,0}^{\mathrm{H}}\mathbf{F}^{\mathrm{H}}\mathbf{D}_{t_{0}+\tau_{m,0}}\mathbf{F}\mathbf{s}\right)\!\right\}\right)^{2}\!. \end{aligned}}  $$

The above steps represent the coherent ML algorithm.

The search for the best values of (*t*_0_,*x,y,z*) must be very fine, but this would result in high numerical complexity. As an alternative, we propose a statistically suboptimal approach but numerically much more efficient. Without loss of generality, we select the first channel to be the reference channel. In a preprocessing step, we estimate the total delay in that channel, *t*_1_=*t*_0_+*τ*_1,0_ from

20 $$ \widehat{t}_{1} = \underset{t_{1}}{\mathrm{arg\,max}}\left(\text{Re}\left(\mathbf{u}_{1,0}^{\mathrm{H}}\mathbf{F}^{\mathrm{H}}\mathbf{D}_{t_{1}}\mathbf{F}\mathbf{s}\right)\right).  $$

This maximization can further be simplified by breaking it down into three steps. First, we estimate an integer-valued delay $\widehat {t}_{1,\text {int}}$, dismissing the carrier phase, from

21 $$ \widehat{t}_{1,\text{int}}= \underset{t_{1,\text{int}}}{\mathrm{arg\,max}}\left|\mathbf{u}_{1,0}^{\mathrm{H}}\mathbf{F}^{\mathrm{H}}\mathbf{D}_{t_{1,\text{int}}}\mathbf{F}\mathbf{s}\right|,  $$

which reduces to

22 $$ \begin{aligned} \widehat{t}_{1,\text{int}}= \underset{t_{1},\text{int}}{\mathrm{arg\,max}} \left|\mathbf{u}_{1,0}^{\mathrm{H}}\left[s(N-t_{1,\text{int}}),\hdots,s(N-1), \right. \right.\\ \left.\left.s(0),\hdots,s(N-t_{1,\text{int}}-1)\right]^{\top}\right|. \end{aligned}  $$

In the second step, we find a fractional, but still a relatively rough estimate $\widehat {t}_{1,\mathrm {r}}$ by searching in a smaller interval, say, $t_{1}\in \left (\widehat {t}_{1,\text {int}}-0.5,\widehat {t}_{1,\text {int}}+0.5\right)$, also dismissing the carrier phase and using (21), or

23 $$ \begin{aligned} \widehat{t}_{1,\mathrm{r}} &= \underset{t_{1,\mathrm{r}}\in {\mathcal{R}}} {\mathrm{arg\,max}}\left|\mathbf{u}_{1,0}^{\mathrm{H}}\mathbf{F}^{\mathrm{H}}\mathbf{D}_{t_{1,\mathrm{r}}}\mathbf{F}\mathbf{s}\right|,\\ {\mathcal{R}}&=\left(\widehat{t}_{1,\text{int}}-0.5,\widehat{t}_{1,\text{int}}+0.5\right).  \end{aligned}  $$

In the third step, we estimate with the highest accuracy $\hat {t}_{1}$, by searching in the smallest interval around $\widehat {t}_{1,\mathrm {r}}$, now relying also on the carrier phase and employing (20).

Finally, once we obtain $\widehat {t}_{1}$, we estimate the location of Tx from

24 $$ {\begin{aligned} (\widehat{x},\widehat{y},\widehat{z})\,=\, \underset{x,y,z}{\mathrm{arg\,max}} \!\!\sum\limits_{m=1}^{M} \! \left(\!\max\!\left\{\!0,\!\text{Re}\!\left(\!\mathbf{u}_{m,0}^{\mathrm{H}}\mathbf{F}^{\mathrm{H}}\mathbf{D}_{\tau_{m,0}-\tau_{1,0}+\widehat{t}_{1}}\mathbf{F}\mathbf{s}\!\right)\!\right\}\!\right)^{2}\!. \end{aligned}}  $$

This is the algorithm we will use in stage 3 of the estimation process. Note that this final search grid does not include the *t*_0_ dimension and that the calculation of the first term in the sum (*m*=1) can be omitted because it is constant. Also, in practice, channel 1 may sometimes have low SNR, and therefore, another channel should be selected as a reference.

One inherent disadvantage of the coherent algorithms is that there are many high and narrow lobes in the criterion function near the true location of the Tx. This is often referred to as the “ambiguity problem.” Stage 3 relies on stage 2 of the localization to correctly identify the main lobe from the side lobes.

Besides the ambiguity problem in the spatial domain, there is also an ambiguity problem in the estimation of *t*_1_ in the time domain. The resulting effect is an additional error which is an integer multiple of 1/*f*_*c*_. This error is equal across the channels and (*x,y,z*). For narrowband signals, its impact on the localization accuracy is negligible. For wideband signals, on average, this error is smaller than for narrowband signals.

#### Non-coherent algorithms

Now, we discuss algorithms that discard carrier phase differences between signals from different channels, unlike the coherent algorithms that exploit these phase differences. The algorithms use the same data as the ones in Section 2.2.1; however, their criterion functions do not fluctuate nearly as much over (*x,y,z*) and as a result their estimates are much less accurate. Convenient consequences of this are that the search grid can be made much coarser and that the ambiguity problem does not exist.

We assume completely unknown phase terms in each channel, *ψ*_*m*,0_, and write

25 $$ \mathbf{s}_{m,0} = a_{m,0}e^{j\psi_{m,0}}\mathbf{F}^{\mathrm{H}}\mathbf{D}_{t_{0}+\tau_{m,0}}\mathbf{F}\mathbf{s}.  $$

We follow the same reasoning as in Section 2.2.1, except that negative values are allowed for *a*_*m*,0_ since we choose the best phase *ψ*_*m*,0_ anyway, and formulate the optimization problem as

26 $$ {\begin{aligned} &\left(\widehat{\psi}_{1,0},\hdots,\widehat{\psi}_{M,0},\widehat{t}_{0},\widehat{x},\widehat{y},\widehat{z}\right) \\&\quad \quad= \underset{\stackrel{\psi_{1,0},\hdots,\psi_{M,0}}{t_{0},x,y,z}}{\mathrm{arg\,max}} \quad \sum\limits_{m=1}^{M} \!\! \left(\text{Re}\!\left(e^{j\psi_{m,0}}\mathbf{u}_{m,0}^{\mathrm{H}}\mathbf{F}^{\mathrm{H}}\mathbf{D}_{t_{0}+\tau_{m,0}}\mathbf{F}\mathbf{s}\right)\right)^{2}\!. \end{aligned}}  $$

We can find the solutions for *ψ*_*m*,0_ separately. Namely, for given *t*_0_, *x*, *y*, *z*, the solutions for *ψ*_*m*,0_ are given by

27 $$ \widehat{\psi}_{m,0} = -\arg\left(\mathbf{u}_{m,0}^{\mathrm{H}}\mathbf{F}^{\mathrm{H}}\mathbf{D}_{t_{0}+\tau_{m,0}}\mathbf{F}\mathbf{s}\right), \;\;\;m=1, 2, \ldots, M.  $$

When these solutions are substituted in (26), we optimize over (*t*_0_,*x,y,z*), or

28 $$  \left(\widehat{t}_{0},\widehat{x},\widehat{y},\widehat{z}\right) =\underset{t_{0},x,y,z}{\mathrm{arg\,max}}\sum\limits_{m=1}^{M} \left|\mathbf{u}_{m,0}^{\mathrm{H}}\mathbf{F}^{\mathrm{H}}\mathbf{D}_{t_{0}+\tau_{m,0}}\mathbf{F}\mathbf{s}\right|^{2}.  $$

We refer to the algorithm based on the solutions in (27) and the optimization in (28) as the noncoherent ML algorithm.

We also propose a noncoherent ML algorithm with reduced computational complexity. As in the coherent algorithm, we first estimate the total delay in channel 1 and use the obtained estimate to search for the coordinates of Tx, or

29 $$ \left(\widehat{x},\widehat{y},\widehat{z}\right) = \underset{x,y,z}{\mathrm{arg\,max}}\sum\limits_{m=1}^{M} \left|\mathbf{u}_{m,0}^{\mathrm{H}}\mathbf{F}^{\mathrm{H}}\mathbf{D}_{\tau_{m,0}-\tau_{1,0}+\widehat{t}_{1}}\mathbf{F}\mathbf{s}\right|^{2}.  $$

This is the algorithm we will use in stage 1 of the estimation process. The first term in the sum is constant, so it can be omitted (the index *m* can take just the values 2,3,…,*M*). Also note that there is no need for the estimate $\widehat {t}_{1}$ to be as accurate as in the implementation of the coherent algorithm. Therefore, one can skip step 3 of the method for estimating *t*_1_ accurately. Instead of $\widehat {t}_{1}$, one can use $\widehat {t}_{1,\mathrm {r}}$.

#### Semi-coherent algorithms

The difference between the coherent and non-coherent algorithms is in the way how the summation in the criterion function is used; in the coherent algorithm, it is the real component of the sum that is applied whereas in the non-coherent algorithm, it is the absolute values of its terms that are exploited (cf. (24) and (29)). By taking absolute values, the constant phase differences among channels are lost. We can use this idea to formulate a semi-coherent algorithm by taking the appropriate absolute values before summing over the channels (over *m*), but after summing over the antennas in each subarray (over *l*). In this way, the phase differences between the antennas of the same subarray are preserved, whereas the phase differences between subarrays are lost.

**Fig. 4:**
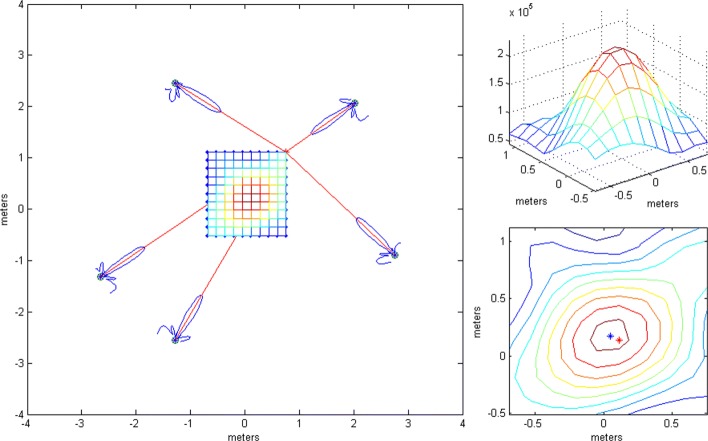
Stage 2a scanning

**Fig. 5:**
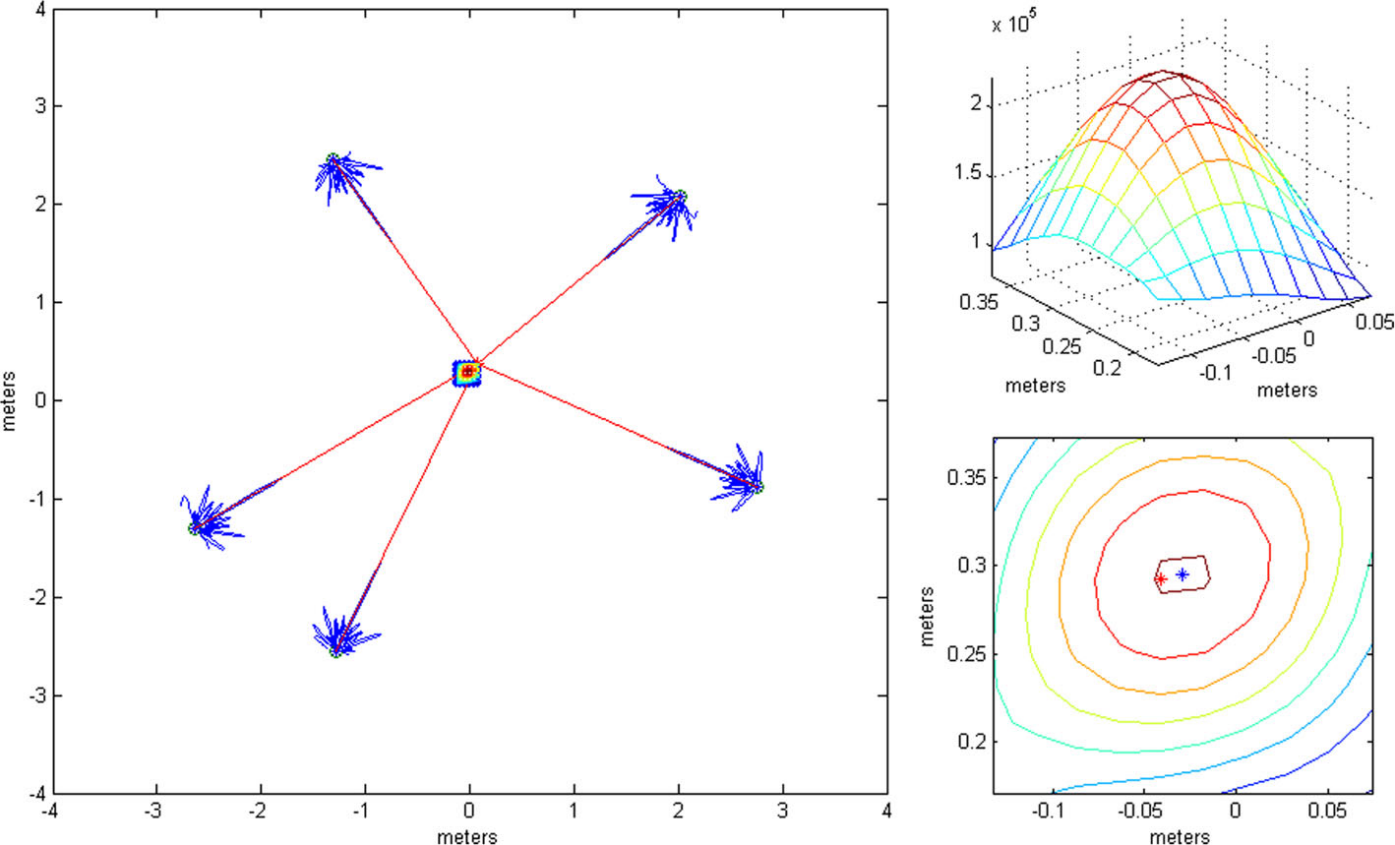
Stage 2b scanning

Let us choose a scan grid inside a given area of interest, see Figs. 4 and 5. Let $\vec {r}_{\text {SG}p}$ be the position of the *p*th point on the scan grid. Unlike the coherent and non-coherent algorithms, for each point on the scan grid, a new *N*-sample-long signal segment, $\mathbf {u}_{m}^{(p)}$, (∀*m*), is received by the beamformers whose beams have been directed to this point. The beams are formed by setting their coefficients *w*_*m,l*_. The 3 dB widths of the beams are chosen to be greater than the distance between adjacent scan-grid points to avoid degradation due to the grid. The objective is to estimate *p* and *t*_0_ by

30 $$ (\widehat{p},\widehat{t}_{0}) = \underset{p,t_{0}}{\mathrm{arg\,max}}\sum\limits_{m=1}^{M} \left|\mathbf{u}_{m}^{(p)\mathrm{H}}\left(\sum\limits_{l=1}^{L_{m}} \mathbf{F}^{\mathrm{H}}\mathbf{D}_{t_{0}}\mathbf{D}^{(\mathrm{B})}_{\tau_{m,l}}\mathbf{F}\mathbf{s}\right)\right|,  $$

where $\mathbf {D}^{(\mathrm {B})}_{\tau } = \text {Diag}\left \{\exp \left (-j\frac {2\pi }{N}\tau \mathbf {k}\right)\right \}$ denotes a baseband signal time delay matrix without a carrier phase shift, unlike **D**_*τ*_. The position estimate of Tx is then $\hat {\vec {r}} = \vec {r}_{\text {SG}\hat {p}}$.

As before, we can avoid estimating *t*_0_ by estimating $\widehat {t}_{m}$ for each omni antenna in preprocessing and then maximizing over *p*, i.e.,

31 $$ \widehat{p} = \underset{p}{\mathrm{arg\,max}}\sum\limits_{m=1}^{M} \left|\mathbf{u}_{m}^{(p)\mathrm{H}}\left(\sum\limits_{l=1}^{L_{m}} \mathbf{F}^{\mathrm{H}}\mathbf{D}_{\hat{t}_{m}-\tau_{m,0}}\mathbf{D}^{(\mathrm{B})}_{\tau_{m,l}}\mathbf{F}\mathbf{s}\right)\right|.  $$

This is the algorithm we will use in stage 2 of the estimation process.

If the signals are narrowband, the expressions (30) and (31) reduce to (32) and (33), respectively:

32 $$\begin{array}{*{20}l} (\widehat{p},\widehat{t}_{0}) & = \underset{p,t_{0}}{\mathrm{arg\,max}} \sum\limits_{m=1}^{M} \left|\mathbf{u}_{m}^{(p)\mathrm{H}}e^{-j\omega_{c} t_{0}}\mathbf{s}\right|, \end{array} $$

33 $$\begin{array}{*{20}l} \widehat{p} & = \underset{p}{\mathrm{arg\,max}} \sum\limits_{m=1}^{M} \left|\mathbf{u}_{m}^{(p)\mathrm{H}}e^{-j\omega_{c} \left(\widehat{t}_{m}-\tau_{m,0}\right)}\mathbf{s}\right|. \end{array} $$

## Numerical results and discussion

This section provides numerical results obtained by the presented algorithms with Monte Carlo simulations for two scenarios—a LoS-only scenario and a multipath scenario. We experimented with two distributed receiver antenna array geometries for each of the two scenarios. Geometry *G*_1_ consists of five antenna subarrays, each subarray having the geometry of an 18-element acoustic camera scaled down by a factor of 3 [39]. One omni antenna is added to the center of each subarray. The centers are in the plane, *z*=0. The omni antennas have the following coordinates *x* and *y* in meters: (−2.20,−1.24), (0.18,−2.64), (2.96,−1.06), (2.53,2.21), and (−2.18,2.24). They are represented by white triangles in the figures in this section.

The positions of the subarrays were chosen by hand in order to be irregular. The distances between subarrays were selected to correspond to subarrays placed on the walls of a room. The subarrays have planar geometry in vertical plains, rotated around their vertical axes so that their broadside directions (approximately) point to the center of the area between subarrays (the room). Geometry *G*_2_ is formed from *G*_1_ by scaling up by a factor of five the antenna positions in the subarrays with respect to their centers (omni antennas). The simulations were carried out using a known deterministic sequence, the first of the modulatable orthogonal sequences proposed in [40] for a given *N*. The parameters were as follows: *ν*_*c*_=60 GHz *B*=10 MHz, and SNR_0_= 10 dB (if not stated otherwise), where SNR_0_ denotes the SNR in a virtual channel whose antenna is at a distance of 1 m from the transmitting antenna. Throughout this section, we assume that the signal power decreases with squared distance from the transmitter.

In the multipath scenario, we simulated a specular multipath. We simulated only first-order reflected paths off four vertical planes (*x*=−2.4 m, *y*=−2.85 m, *x*=3.15 m, and *y*=2.45 m), which represented walls of a room, so that the subarrays are attached to them (at a distance of about 20 cm). As in [41], we assumed a ratio of LoS component power and the sum of reflected component powers (the Rice factor) of at least 10 dB. According to the ray-tracing method, the reflected components were modeled as if they were sent by virtual images of the Tx (w.r.t. the walls) according to the LoS model (7) (which includes a time shift, a carrier phase shift, and an attenuation). The reflected components were then phase shifted by *π*, and their sum at each receiving antenna was scaled to get the specified Rice factor.

### Qualitative characterization of the criterion functions

**Fig. 6:**
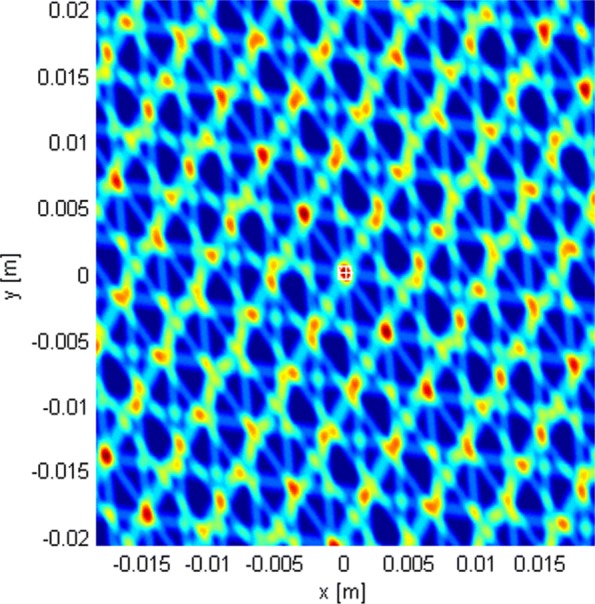
The criterion function of stage 3, given by (24) with *N*=1024

**Fig. 7:**
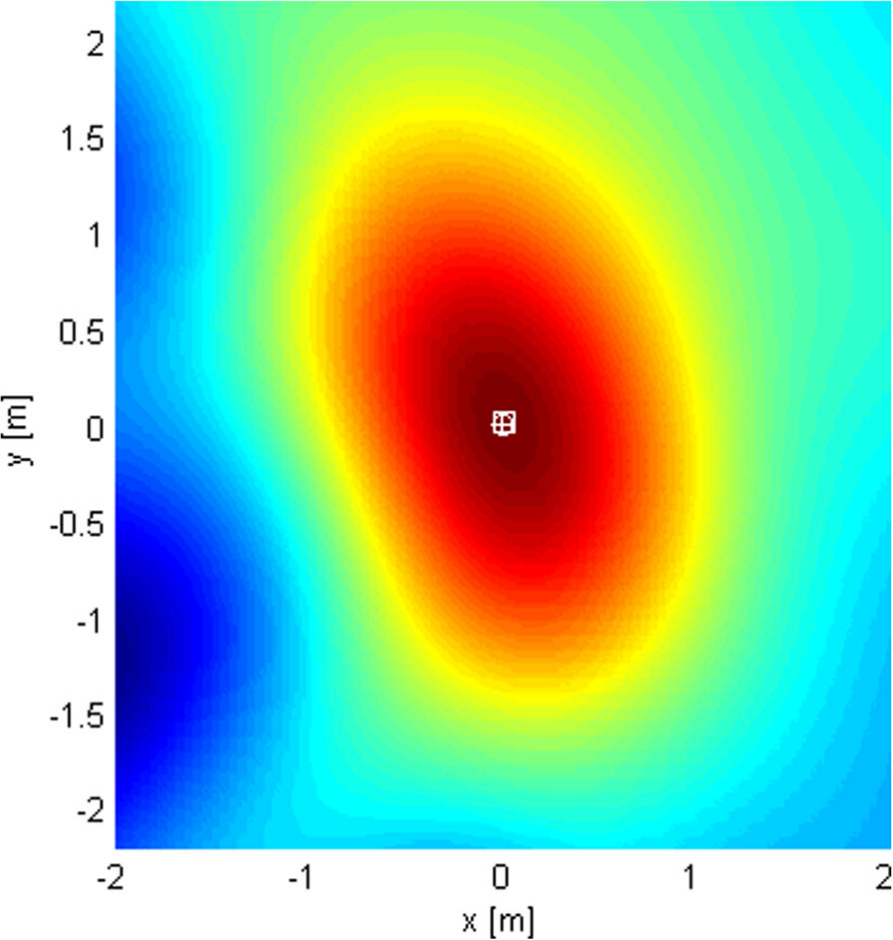
Criterion function of stage 1, given by (29) with *N*=1024

**Fig. 8:**
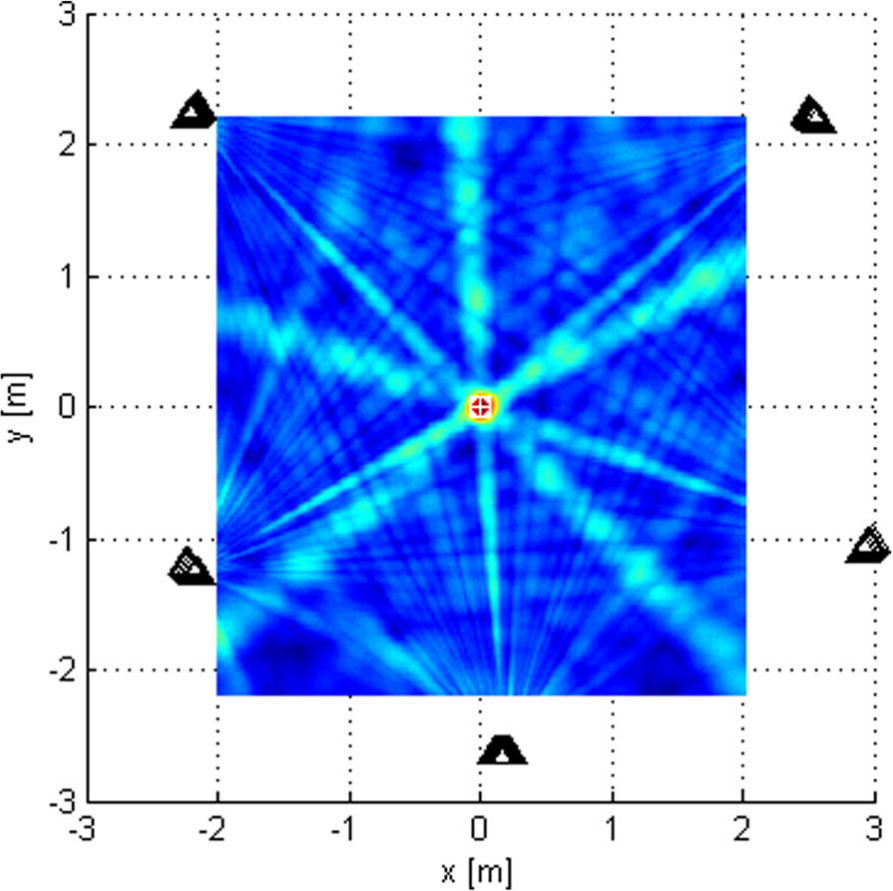
Criterion function of stage 2, given by (31) for *G*_1_ with *N*=1024

**Fig. 9:**
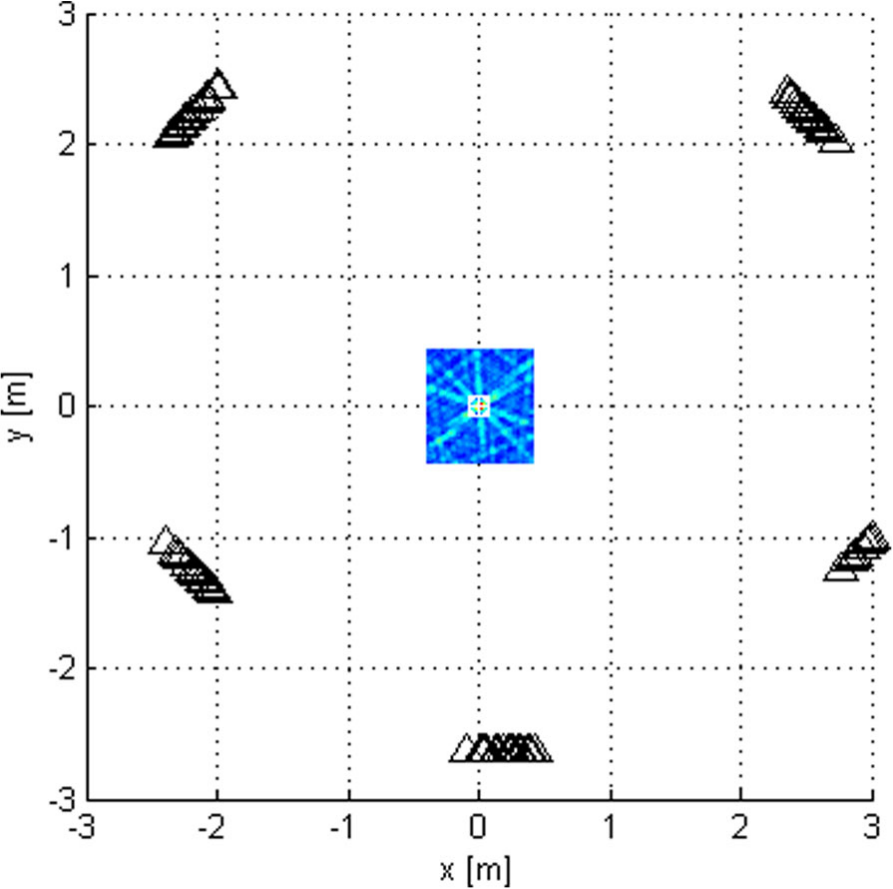
Criterion function of stage 2 for *G*_2_ with *N*=1024

**Fig. 10:**
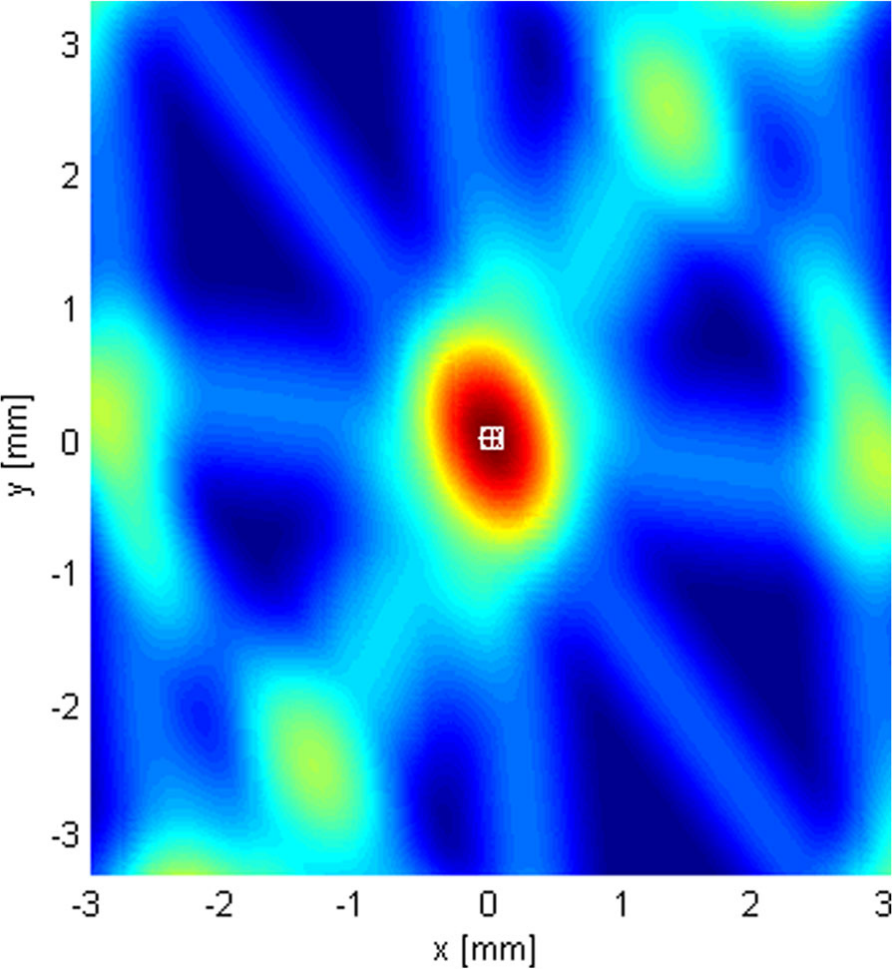
Criterion function of stage 3, given by (24) with *N*=1024

**Fig. 11:**
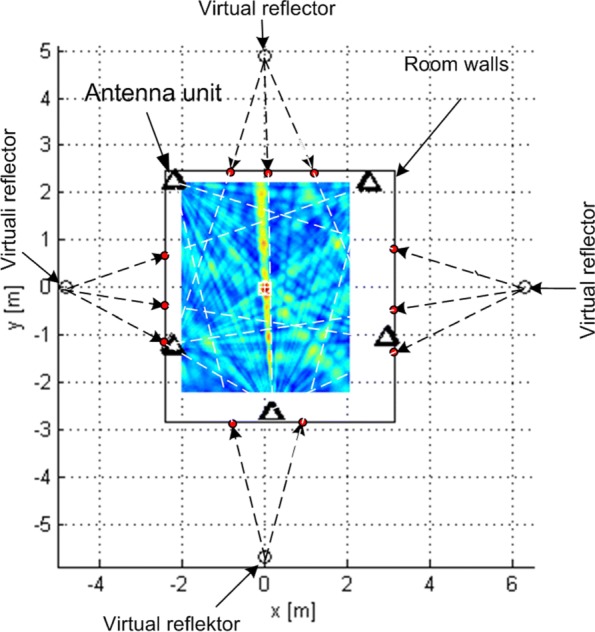
Criterion function for stage 2, *G*_1_, Rice factor 10 dB, and *N*=1024 with ray-tracing

This subsection shows the qualitative behavior of the criterion functions of the respective algorithms for each of the three localization stages. The Tx was at (0,0,0), near the “center” of either *G*_1_ or *G*_2_. Since stages 1 and 3 employ only omni antennas, the choice of subarray geometry does not matter. The criterion functions are shown over areas lying in the plane *z*=0. In Figs. 6, 7, 8, 9, 10, and 11, the true Tx location is marked by a circle with a cross and the estimated location by a square.

In order to localize the Tx with accuracy much better than *λ*_*c*_, we use the coherent algorithm. Its criterion function has high side lobes and requires a very fine search grid (see Fig. 6). Therefore, we cannot work with it immediately, but instead we resort to a multistage/multiresolution search.

Figure 7 shows the LoS-only criterion function of stage 1 (given by (29)) over an area inside the antenna array for *N*=1024. The function does not have side lobes, it is not influenced by carrier phases, it is immune to phase synchronization errors, and it varies slowly across space, which suggests that a coarse grid can be used.

Figure 8 shows the LoS-only criterion function of stage 2 over an area inside the antenna array for *G*_1_ for *N*=1024. We have also generated the corresponding criterion function for *N*=16, but it is not shown because it has the same shape. It also has no side lobes but shows more variations across space compared to the criterion function of stage 1. This function offers better estimation accuracy. Figure 9 shows the same results over a smaller area for geometry *G*_2_. The figures suggest that the plane wave assumption would not be justified because of the size of subarray apertures.

Figure 10 shows the LoS-only criterion function of stage 3 for *N*=1024 over an area around the transmitter spanning a little more than the main lobe. As the used algorithm is coherent (utilizes information in the phase of the carrier for localization), the criterion function has side lobes, separated by approximately 2*λ*_*c*_/3. Since we use an adaptive search grid in this stage, the algorithm finds the peak of the lobe it has been initialized on (the initialization point is the estimate obtained in stage 2). Clearly, to prevent the algorithm from converging to a side lobe, the localization in stage 2 must produce an estimate inside the main lobe of the criterion function of stage 3. In other words, if the localization error of stage 2 is smaller than approximately *λ*_*c*_/3, the ambiguity problem is resolved, because the displacement of the center of the main lobe in stage 3 due to noise is small compared to its width without noise.

Figure 11 shows the criterion function of stage 2 in the multipath scenario for *N*=1024 over an area inside the antenna array for *G*_1_, along with wall positions and the “rays” from the ray-tracing method. The Rice factor was 10 dB. The figure shows that the localization algorithm is robust w.r.t. the multipath propagation since the lobes corresponding to the reflected rays cannot be seen. The criterion functions for stage 1 and 3 with multipath propagation are not given because they are almost identical to the LoS-only ones.

### Quantitative characterization of the algorithms

In this subsection, we evaluate the accuracy of the algorithms of each stage. As performance metrics, we used both the MSE (mean squared error) and RMSE of the estimates.

**Fig. 12:**
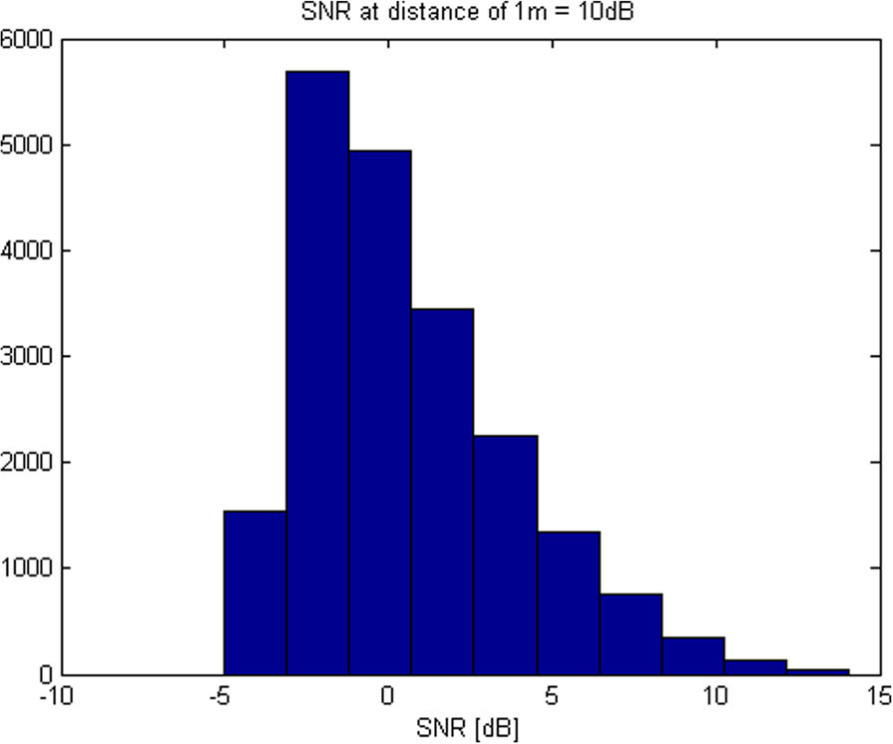
Distribution of SNRs at the antennas for simulated Tx locations for SNR_0_=10 dB

In Fig. 12, we display the histogram of the SNRs at the antennas of array *G*_1_ (it is similar for *G*_2_) for all simulated transmitter locations (points of the Tx grid) for SNR_0_=10 dB. Most of the SNRs are between − 5 and 10 dB.

The contour plots in the rest of the section were generated over a Tx grid of 16×16 points that covers most of the area inside the array to show the error distribution across space. The grid has uniformly spaced points in the plane *z*=0.

**Fig. 13:**
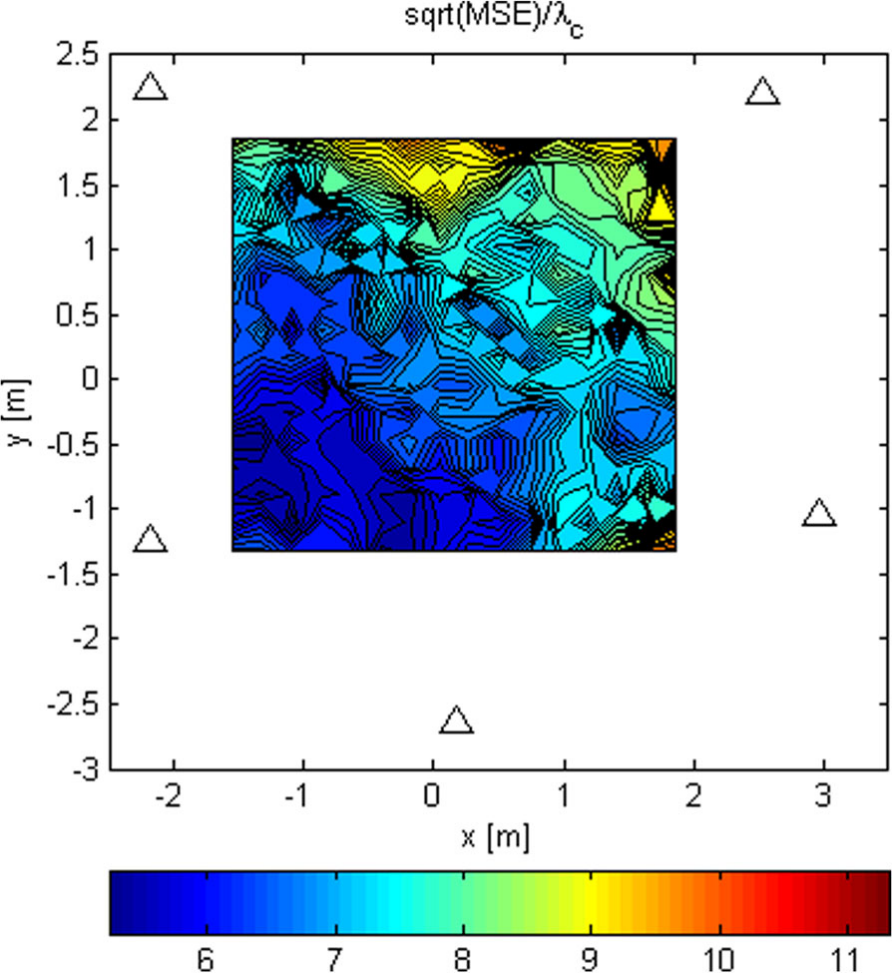
RMSE/ *λ*_*c*_ for stage 1 and *N*=1024

In Fig. 13, we plotted the LoS-only RMSEs relative to the carrier wavelength, *λ*_*c*_, for stage 1 for *N*=1024. For every Tx grid point, we performed 100 Monte Carlo runs and averaged out the results. Note that the accuracy is generally better near the first antenna because it is used as the reference antenna for estimating *t*_1_. If the position estimate of this stage is far away from the reference antenna, another antenna can be adopted as the reference and the process is repeated. Stage 3 could benefit from choosing a better reference antenna even more. The RMSE of stage 1 varies between 6 and 12 *λ*_*c*_ in the given area. These values determine how narrow the search grid in the next stage can be for a given Tx position, because the grid should include the real Tx position. If the estimation errors have Gaussian distributions, the search grid for the next stage should span an area that is ± 2 standard deviations of the current stage along each dimension in order to include the real location with probability of 0.95.

**Fig. 14:**
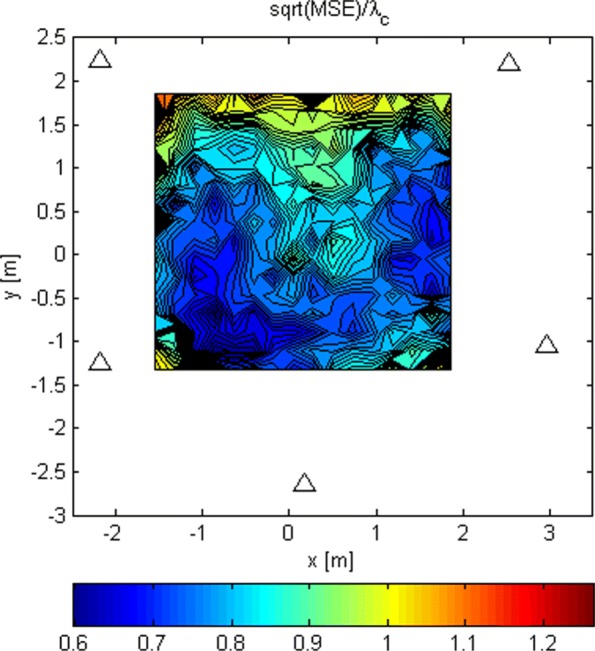
RMSE/ *λ*_*c*_ of stage 2 for *G*_1_ and *N*=1024

**Fig. 15:**
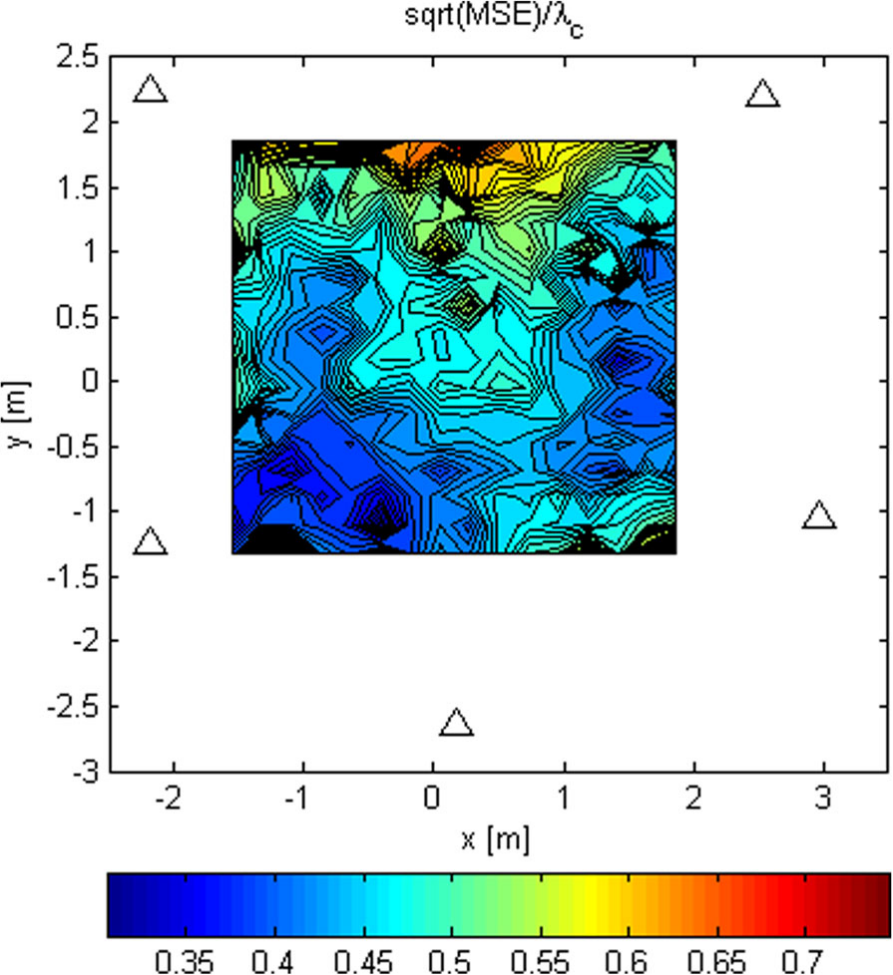
RMSE/ *λ*_*c*_ for stage 2 for *G*_2_ and *N*=16

**Fig. 16:**
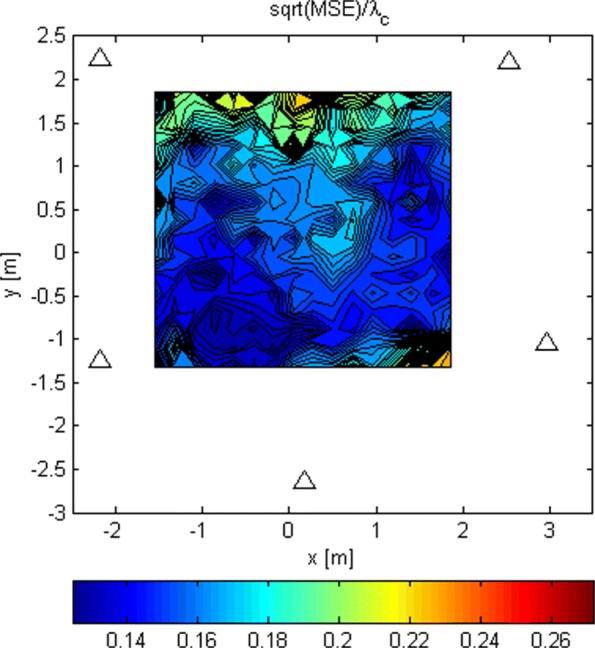
RMSE/ *λ*_*c*_ of stage 2 for *G*_2_ and *N*=1024

The LoS-only RMSEs relative to *λ*_*c*_ for stage 2 for *G*_1_ and *N*=1024 is shown in Fig. 14. Again, we ran 100 Monte Carlo simulations for every Tx grid point and computed from them the RMSEs. The same results but for *G*_2_, and *N*=16 and *N*=1024, are presented in Figs. 15 and 16. These results are better because of the increased space between the antennas in the subarrays. The increased space produces “narrower beams,” i.e., better spatial selectivity. For *N*=1024 and *G*_2_, the RMSE is below *λ*_*c*_/6 over a significant part of the area inside the array. This allows the search in stage 3 to start somewhere within the main lobe of its criterion function with a probability of 0.95. Thus, we can argue that the ambiguity problem is avoided with high probability. The simulations in which the analog beamformers had phase quantization with a resolution of 3° were also carried out. The results are not shown here because they were almost identical to the ones without phase quantization.

**Fig. 17:**
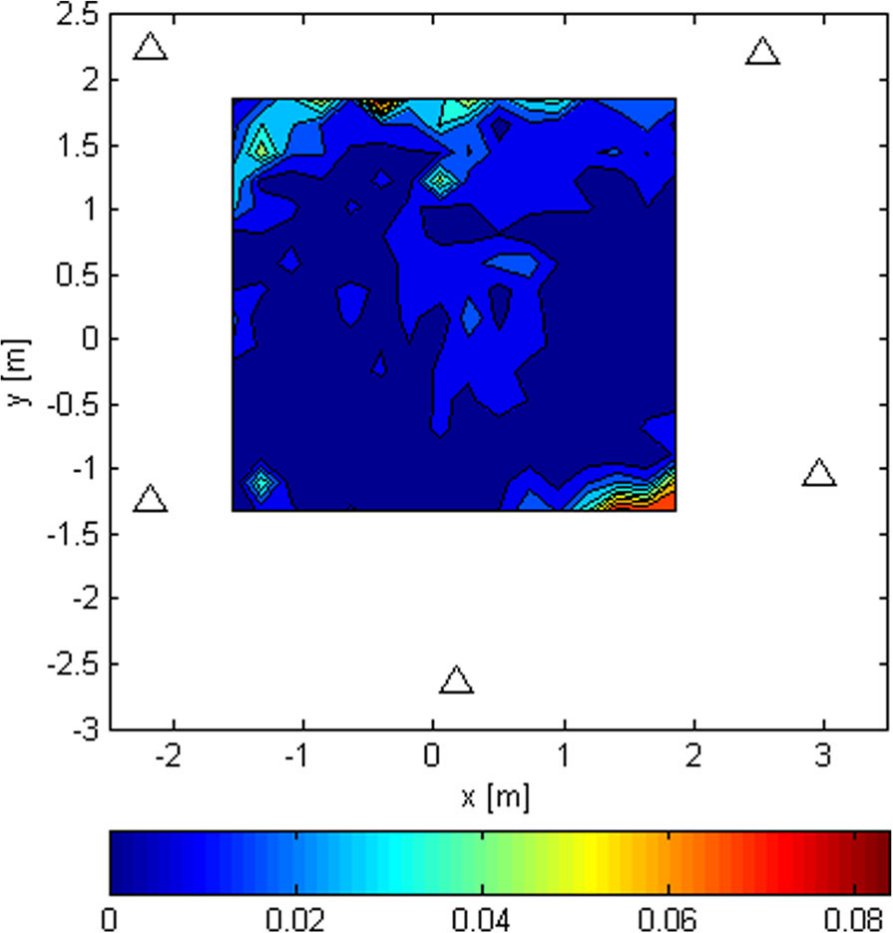
Probability of missing the main lobe in stage 3 due to the error in stage 2, for *G*_2_ used in stage 2 and *N*=1024

**Fig. 18:**
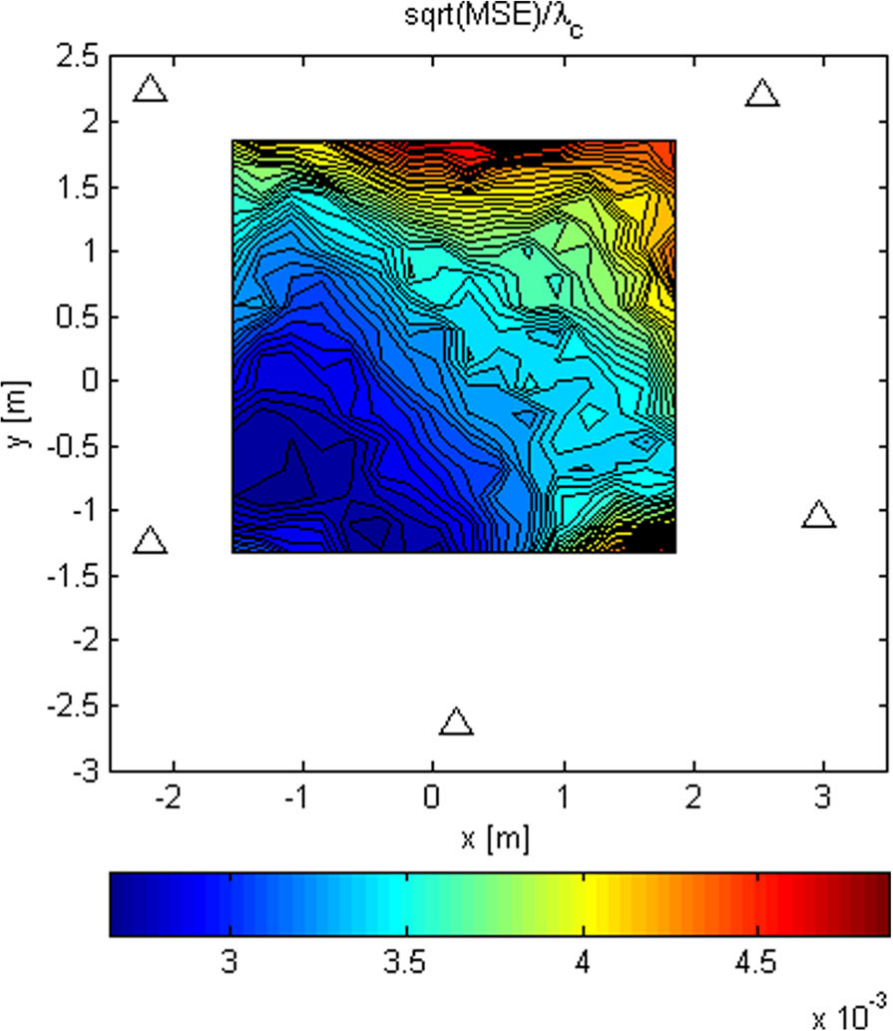
RMSE/ *λ*_*c*_ of stage 3 and *N*=1024 when the main lobe is not missed

**Fig. 19:**
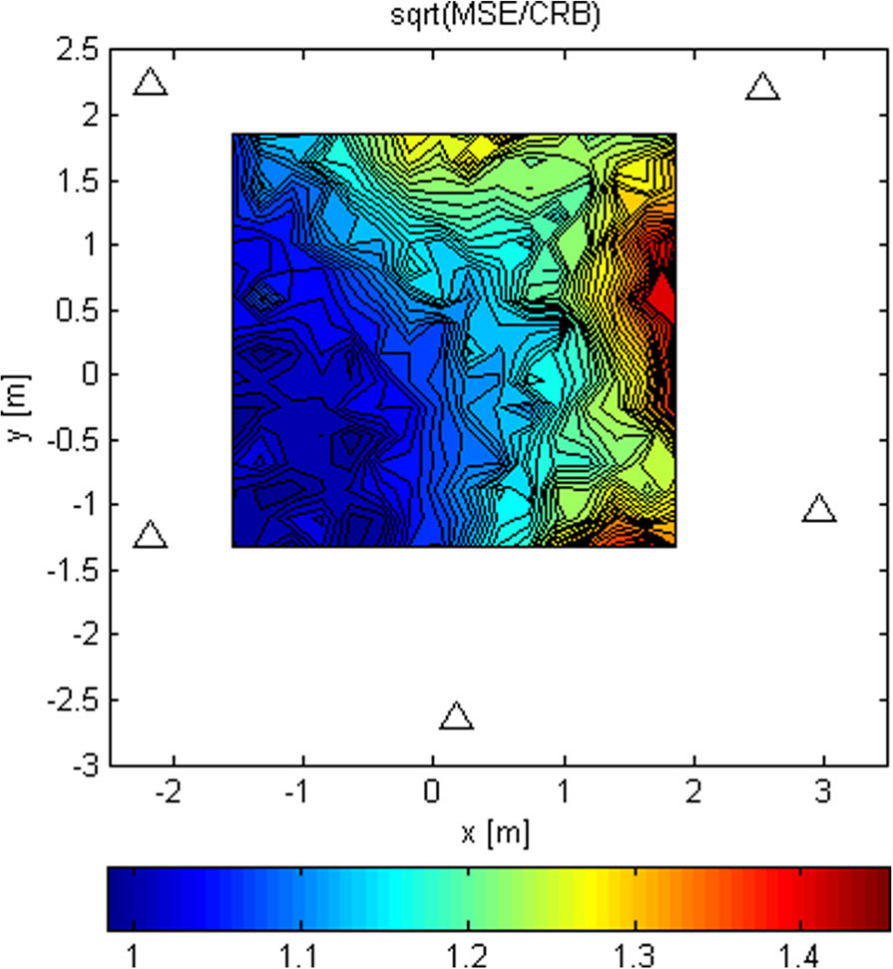
MSE/CRB of stage 3 and *N*=1024 when the main lobe is not missed

**Fig. 20:**
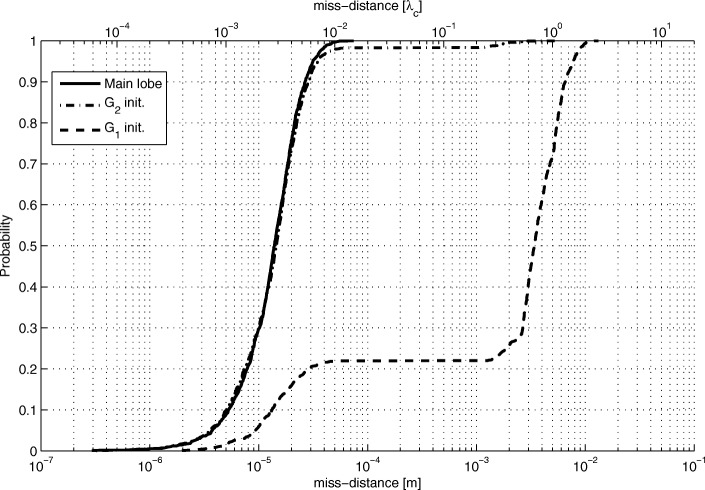
Stage 3 error CDF curves for different initializations

The LoS-only results for stage 3 for *N*=1024 are plotted in Figs. 17, 18, and 19. In this experiment, for every Tx grid point, we had 1000 Monte Carlo runs. In Fig. 17, we see how the probability of obtaining an estimate from a side lobe (or that the main lobe was missed) varies across the Tx grid. This probability depends on the estimate of stage 2 because the algorithm of stage 3 uses an adaptive grid that converges to the maximum of the lobe on which it has been initialized. Figure 18 shows the RMSE across the Tx grid provided that the main lobe has not been missed. As for stage 1, the effect of choosing the reference antenna can be seen (because the accuracy is better near the reference antenna). In this case, the obtained accuracy is of the order of *λ*_*c*_/100. For comparison reasons, we have also run simulations for SNR_0_=20 dB, and the RMSE for the Tx at (0,0,0) is *λ*_*c*_/963. In Fig. 19, we observe the statistical efficiency measured as the ratio of MSE and Cramér-Rao bound for the stage 3 algorithm when the main lobe has not been missed. Figure 20 shows LoS-only CDF (cumulative density function) curves of the stage 3 localization error for *N*=1024 for three cases: (1) the main lobe is not missed, (2) the stage 3 algorithm is initialized by the results of the stage 2 algorithm for *G*_2_ (see Fig. 16), and (3) the stage 3 algorithm is initialized by the results of the stage 2 algorithm for *G*_1_ (see Fig. 14). The CDF curves were obtained for the same Tx grid as for the contour plots (e.g., Fig. 13) with five runs for each grid point. As predicted, the algorithm in case (2) misses the main lobe only 2% of the time. On the other hand, the algorithm in case (3) misses the main lobe 78% of the time.

For easier comparison of the numerical results for the LoS-only scenario, we provide them in Table 1. The first row shows the RMSE averaged over the Tx grid, the second row the RMSE at a point near the center of the array (roughly (0,0,0)), and the third row the value not exceeded by the RMSE of 80% of the points on the Tx grid. The results are for the case when the main lobe in stage 3 is not missed.

**Table 1:** RMSEs for search/scan stages (*G*_2_ and *N*=1024)

	Stage 1	Stage 2	Stage 3
avg. RMSE	7.56*λ*_*c*_ (37.8 mm)	0.162*λ*_*c*_ (0.81 mm)	0.00365*λ*_*c*_ (0.0182 mm)
cent. RMSE	6.74*λ*_*c*_ (33.7 mm)	0.16*λ*_*c*_ (0.8 mm)	0.00317*λ*_*c*_ (0.0159 mm)
80% RMSE	8.61*λ*_*c*_ (43.1 mm)	0.176*λ*_*c*_ (0.88 mm)	0.00419*λ*_*c*_ (0.021 mm)

**Fig. 21:**
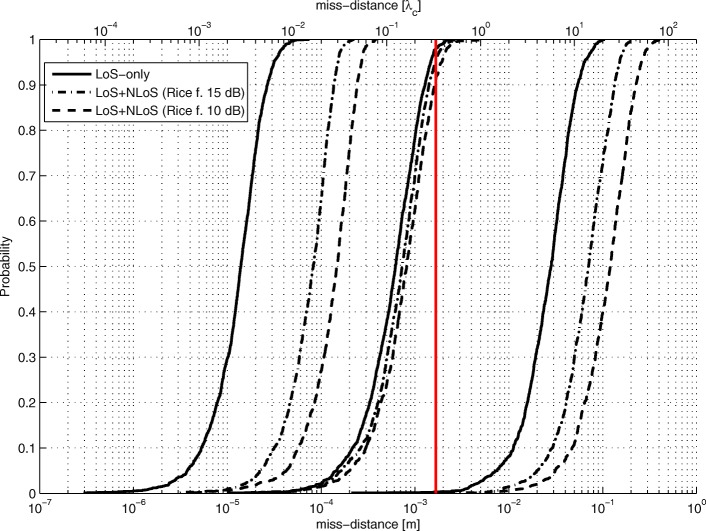
CDF curves of localization errors for different stages and Rice factors for SNR_0_=10 dB

Figure 21 shows CDF curves for different Rice factors for all three localization stages for *N*=1024. For each stage, we show a CDF curve for LoS-only and Rice factor values of 15 and 10 dB. Geometry *G*_2_ was used (in stage 2). Again, these results hold for outcomes when the main lobe in stage 3 is not missed. In stage 1, compared to the LoS-only curve, the error is increased roughly 2.5 and 4.3 times for Rice factors 15 and 10 dB, respectively. In stage 2, the error is increased 1.2 and 1.3 times. In stage 3, the error increase is 6 and 10 times. Stage 2 is the least affected by multipath propagation thanks to the beam directivity of the subarrays. The vertical line at *λ*_*c*_/3 shows whether the stage 2 estimate is within the main lobe of stage 3 or not. This is a critical value for solving the ambiguity problem. Even for a Rice factor of 10 dB, the ambiguity problem is solved 90% of the time.

**Fig. 22:**
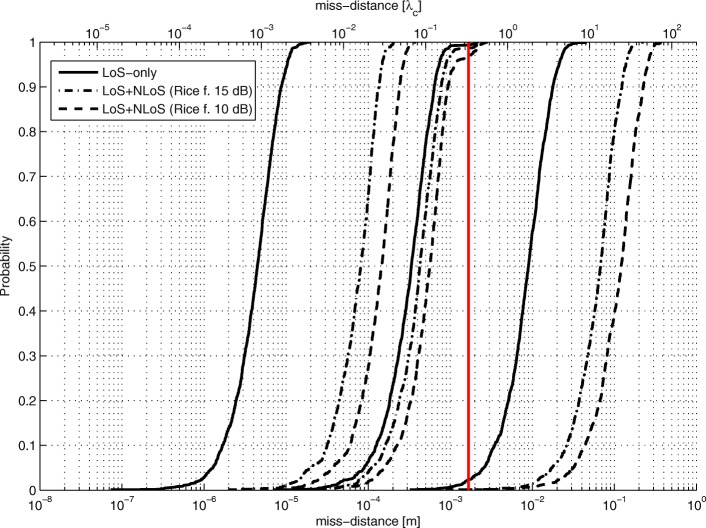
CDF curves of localization errors for different stages and Rice factors for SNR_0_=20 dB

Figure 22 shows the appropriate CDF curves for SNR_0_=20 dB (instead of 10 dB as in Fig. 21). As expected, the results for the LoS-only scenario are better. However, the results for the multipath environment are practically the same. Therefore, the adverse effect of multipath propagation is greater for higher SNR values.

To summarize, as opposed to the existing methods mentioned in Section 1, which achieve a submeter localization accuracy, the proposed methods improve that accuracy to a small fraction of the carrier wavelength, which enables the shift from location-based services to location-based communication for dramatic improvement of a 5G system performance.

## Conclusions

In this paper, we addressed indoor position estimation with a millimeter-wave massive MIMO system. We proposed an architecture with distributed antenna units, a multistage/multiresolution strategy, and three classes of localization algorithms that together achieve RMSE of up to three orders better than the carrier wavelength, and solve the ambiguity problem, inherent to coherent algorithms. In the LoS-only scenario, the localization error is by two to three orders better than the carrier wavelength, whereas in the specular multipath scenario, it is up to 10 times worse for realistic Rice factors, but still well below the carrier wavelength. The strategy does not require channel-state information and is applicable in multi-user scenarios, but requires dominant LoS propagation. The studied signal model is inherently wideband, and it assumes spherical wavefronts. The execution of the algorithms can be partially distributed among the subarray units. The obtained accuracy allows the base station array to focus energy to the position of the localized user terminal on downlink and to receive its uplink signal emitted with decreased power. This can dramatically improve the overall capacity of the millimeter-wave massive MIMO system. An open issue is positioning of the BS antennas with accuracy greater than that of the localization, including the orientation of the subarrays.

## Abbreviations

3GPP: 3rd Generation Partnership Project
5G: 5th generation
A/D: Analog-to-digital
BS: Base station
CDF: Cumulative density function
DoA: Direction of arrival
CRB: Cramér-rao bound
D/A: Digital-to-analog
DFT: Discrete Fourier transform
DSP: Digital signal processor
IQ: In-phase quadrature-phase
LoS: Line-of-sight
MIMO: Multiple-input-multiple-output
ML: Maximum likelihood
MSE: Mean squared error
NLoS: Non-line-of-sight
PDF: Probability density function
RF: Radio frequency
RFID: Radio frequency identification
RMSE: Root mean squared error
RSS: Received signal strength
RSSI: Received signal strength indicator
SNR: Signal-to-noise-ratio
ToA: Time of arrival
Tx: Transmitter
UT: User terminal
